# Acupotomy for adhesive capsulitis of the shoulder: A case report

**DOI:** 10.1097/MD.0000000000044115

**Published:** 2025-08-22

**Authors:** Zehao Hu, Qiuxuan Huang, Zhanxin Li

**Affiliations:** a Shantou Hospital of Traditional Chinese Medicine, Guangzhou University of Traditional Chinese Medicine, Shantou, China; b Graduate School of Guangzhou University of Traditional Chinese Medicine, Guangzhou, China; c Zhanxin Acupotomology Research Institute, Foshan, China.

**Keywords:** acupotomy therapy, adhesive capsulitis of the shoulder, biomechanics, case report, minimally invasive techniques

## Abstract

**Rationale:**

Adhesive capsulitis of the shoulder (ACS) is a pathological condition characterized by chronic inflammation and fibrosis of the glenohumeral joint capsule and surrounding soft tissues, leading to capsular adhesion and stiffness. Its hallmark clinical manifestations include progressive pain and restricted active/passive range of motion. Acupotomy therapy, which integrates traditional Chinese meridian tendon theory with modern biomechanical principles, achieves minimally invasive adhesion release and biomechanical balance restoration. This article presents a successfully treated case of adhesive shoulder capsulitis using acupotomy.

**Patient concerns:**

The patient, a 52-year-old male, presented with persistent pain in the left shoulder accompanied by restricted joint mobility and evident muscle atrophy.

**Diagnoses:**

The patient was diagnosed with ACS on November 15, 2024.

**Interventions:**

Acupotomy was performed to release adhesions in the glenohumeral joint capsule, coracohumeral ligament, and periarticular muscle groups, followed by postoperative joint mobilization and resistance training.

**Outcomes:**

Therapeutic outcomes were evaluated using range of motion, Visual Analog Scale (VAS) for pain, and imaging studies. Immediately after treatment, passive abduction improved to 150° with the VAS score decreasing from 7 to 5; at the 6-week follow-up, active abduction had recovered to 160° with the VAS score stabilized at 2.

**Lessons:**

Acupotomy therapy provides a safe and effective solution for moderate-to-severe frozen shoulder through its stepwise intervention protocol of “adhesion release-dynamic stabilization-functional remodeling,” which restores shoulder biomechanical homeostasis in a minimally invasive manner.

## 1. Introduction

Adhesive capsulitis of the shoulder (ACS), commonly referred to as “frozen shoulder,” is a prevalent condition characterized by chronic inflammation and fibrosis of the glenohumeral joint capsule, along with extensive adhesion of periarticular soft tissues. The disorder typically manifests as shoulder pain, significantly restricted range of motion (ROM), and joint dysfunction, substantially impairing patients’ activities of daily living and quality of life.^[1]^ Epidemiological studies indicate^[2]^ that adhesive capsulitis has an incidence rate of approximately 2% to 5%, with peak prevalence occurring in individuals aged 50 to 60 years. The condition demonstrates a female predominance and shows significant association with diabetes mellitus,^[3]^ thyroid disorders,^[4]^ and prolonged shoulder immobilization. Recent investigations into its pathological mechanisms reveal^[5]^ that chronic nonbacterial inflammatory responses in the shoulder joint capsule and surrounding ligaments/tendons trigger synovial hyperplasia and abnormal collagen deposition, ultimately leading to reduced joint cavity volume and glenohumeral dynamic mechanical imbalance. These pathological changes constitute the fundamental basis for joint stiffness and pain characteristic of this condition.

It is noteworthy that in the differential diagnosis of ACS, it is crucial to clearly distinguish ACS from other diseases that may cause similar shoulder pain and limited range of motion. In addition to the pathological changes of ACS itself, myofascial trigger points (MTrPs) often exist in the periarticular muscles of the shoulder joint (such as trapezius, supraspinatus, subscapularis, etc). These trigger points are highly sensitive sites within the taut bands of skeletal muscles, which can produce characteristic local pain and referred pain when compressed. Their referred pain patterns often overlap with the pain areas of ACS, and similarly lead to limited mobility and functional impairment.^[6]^ Therefore, when evaluating patients with suspected ACS, the peri-shoulder muscle groups should be carefully palpated to screen for MTrPs, which is of key significance for formulating precise treatment plans (such as whether specific interventions for MTrPs are required).

Acupotomy therapy, as an innovative treatment modality integrating traditional Chinese meridian tendon theory with modern minimally invasive techniques, demonstrates significant clinical value in managing adhesive capsulitis of the shoulder. The therapeutic mechanism involves precise release of capsular and periarticular adhesions, elimination of inflammatory entrapment, and restoration of glenohumeral joint biomechanical balance. Concurrently, it stimulates local tissue repair and improves microcirculation, thereby interrupting the vicious cycle of “pain-immobilization-adhesion.”^[7]^ Building upon the pathological correlation between dynamic mechanical imbalance and soft tissue fibrosis in shoulder joints, this article systematically elucidates acupotomy’s intervention mechanisms in regulating inflammatory microenvironments and reconstructing shoulder biomechanical homeostasis. Furthermore, it optimizes treatment parameters and operational pathways to provide evidence-based support and standardized protocols for enhancing clinical outcomes in periarthritis treatment.

## 2. Case presentation

Mr. Zhang, a 52-year-old male, presented for initial consultation on November 15, 2024, with a chief complaint of “left shoulder pain accompanied by restricted mobility persisting for over 5 months.” The patient reported experiencing unexplained onset of left shoulder pain 5 months prior, which worsened nocturnally and progressively led to significant functional impairment in shoulder abduction, adduction, and extension. These limitations rendered him incapable of performing daily activities such as hair combing and belt fastening. Self-administered thermotherapy and oral nonsteroidal anti-inflammatory drugs (NSAIDs) provided no substantial relief. During the past month, mild atrophy of the left deltoid muscle became apparent, prompting his visit to our institution. Physical examination revealed: active left shoulder range of motion limited to <35° abduction, <50° forward flexion, <8° extension, 10° internal rotation, and 10° external rotation, with a visual analog scale (VAS) score of 7. Palpation elicited marked tenderness (++) at the subacromial space, coracoid process, and posterior glenohumeral joint. Passive mobilization elicited characteristic “end-feel resistance” accompanied by articular crepitus, without radiating pain or sensory abnormalities in the upper extremity. Comparative measurements showed 1.2 cm reduction in left deltoid circumference versus the contralateral side, with scapulohumeral muscle strength graded IV. Radiographic findings demonstrated mild left glenohumeral joint space narrowing with inferior humeral osteophyte formation. Diagnosis: left adhesive capsulitis of the shoulder. The treatment protocol comprised acupotomy release of capsular and periarticular adhesions, combined with manual mobilization and functional rehabilitation exercises. Procedure specifications: disposable 0.6 × 50 mm Hanzhang-brand acupotomes were employed. Following comprehensive risk-benefit disclosure, the patient provided written informed consent.

**Figure 1.**
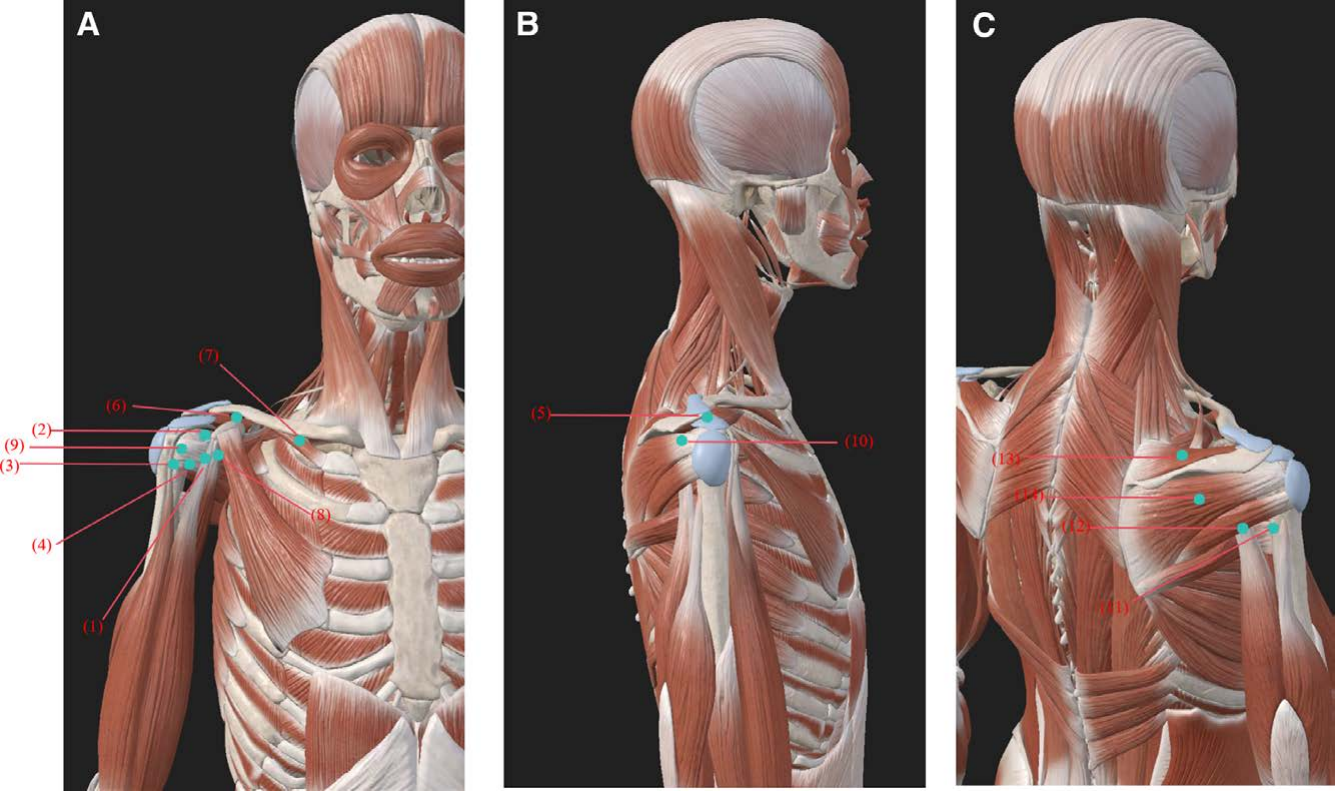
Anatomical location of the operating points of the shoulder joint. (1) Anterosuperior glenohumeral capsule; (2) coracohumeral ligament insertion zone; (3) rotator interval; (4) subscapularis tendon insertion at lesser tubercle; (5) posterior joint capsule; (6) anterior deltoid; (7) clavicular head of pectoralis major; (8) coracobrachialis; (9) long head of biceps tendon; (10) posterior deltoid; (11) conjoined insertion of latissimus dorsi and teres major; (12) long head of triceps; (13) supraspinatus; and (14) infraspinatus. Source: www.3Dbody.com.

Preoperative preparation protocol: The patient was placed in the lateral decubitus position with the left shoulder naturally relaxed and fully exposed. The operator wore standard sterile gloves, cap, and mask. The periarticular skin was disinfected 3 times with povidone-iodine, covering the distal clavicle, acromion, scapular spine, and proximal third of the upper arm. Based on surface anatomy and palpatory landmarks, the following key release points were marked (see Fig. 1): anterosuperior glenohumeral capsule (1 cm inferolateral to the coracoid process); coracohumeral ligament insertion zone (lateral border of the coracoid process); rotator interval (medial to the long head of biceps tendon); subscapularis tendon insertion at lesser tubercle; posterior joint capsule (space between posteroinferior acromion and humeral head); anterior deltoid (anterior border of lateral clavicular third); clavicular head of pectoralis major (anterior midclavicular attachment); coracobrachialis (anteromedial coracoid body); long head of biceps tendon (intertubercular groove); posterior deltoid (inferior scapular spine attachment); conjoined insertion of latissimus dorsi and teres major (posterior axillary line); long head of triceps (infraglenoid tubercle attachment); supraspinatus (mid-portion of supraspinous fossa); infraspinatus (mid-portion of infraspinous fossa).

Operational protocol with technical specifications: The procedure was systematically performed as follows: anterosuperior glenohumeral capsule release: the operator held the acupotome (blade parallel to coracohumeral ligament fibers) and vertically inserted it 1 cm inferolateral to the coracoid process, advancing 2.5 to 3.0 cm to the capsular layer. Four longitudinal cuts were made, followed by lifting-thrusting maneuvers to lyse thickened capsule until tissue elasticity was restored. coracohumeral ligament release: The blade was obliquely inserted to the coracoid’s lateral surface (parallel to ligament axis), performing 3 fan-shaped dissections to relieve contracture. Rotator interval decompression: Entry medial to the biceps long head tendon (blade parallel to humeral axis) allowed longitudinal canalization and transverse oscillation to release peritendinous adhesions. Subscapularis insertion release: two longitudinal cuts were made at the lesser tubercle (blade aligned with tendon fibers) until improved tendon glide was achieved. Posterior capsular release: with the patient slightly forward-leaning, the blade entered the posteroinferior acromion-humeral gap (parallel to joint space) for 3 longitudinal cuts and fan-shaped adhesiolysis. anterior deltoid release: from the anterolateral clavicular junction, 3 longitudinal canalizations were performed along muscle fibers to release fascial restrictions. pectoralis major clavicular head release: A 15°-angled blade performed 2 fan-shaped dissections at the midclavicular attachment to reduce anterior tension. coracobrachialis release: The blade inserted 1 cm medial to the coracoid tip (parallel to muscle axis) delivered 2 longitudinal cuts with transverse oscillation at 2.0 cm depth. Biceps long head tendon release: Three longitudinal cuts in the intertubercular groove sheath reduced tendon sliding resistance. Posterior deltoid release: entry 1 cm lateral to the scapular spine’s inferior border (blade parallel to spine) allowed 3 transverse fanning maneuvers. Latissimus dorsi/teres major conjoined tendon release: A 30°-angled blade at the posterior axillary line performed combined longitudinal and fan-shaped dissection. Triceps long head release: Two longitudinal cuts at the infraglenoid tubercle restored tendon elasticity. Supraspinatus release: mid-supraspinous fossa entry (blade angled superolaterally) delivered 3 longitudinal cuts to address rotator interval adhesions. Infraspinatus release: The acupotome is inserted from the midpoint of the infraspinous fossa of the scapular spine, with the blade line parallel to the muscle fibers. It penetrates into the muscle belly to a depth of 2.0 to 2.5 cm, performing 3 longitudinal loosening maneuvers. If necessary, transverse oscillations are applied to release the contracted fascia and adhesions of the posterior joint capsule.

**Figure 2.**
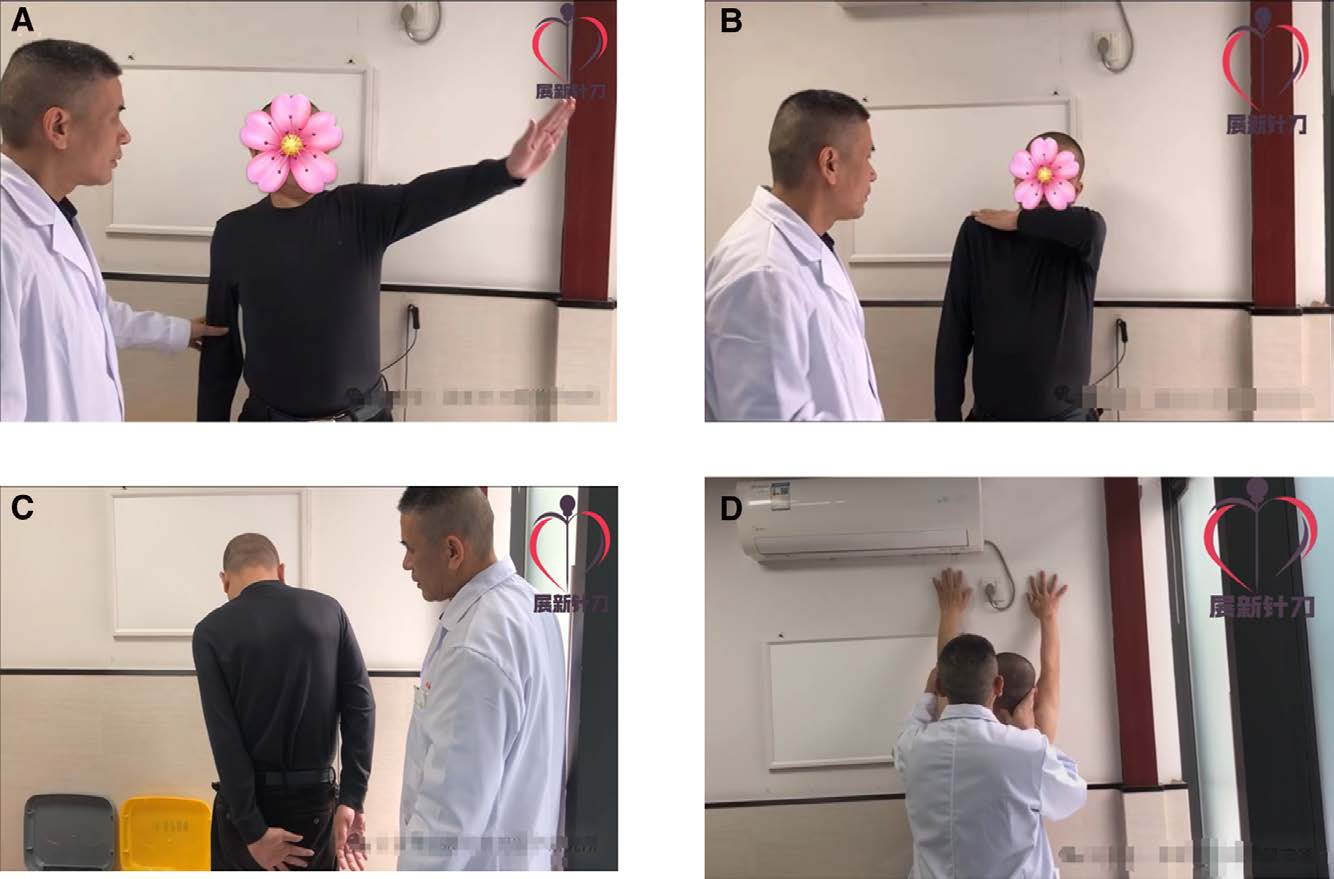
(A–C) Before treatment. (D) After treatment.

Following acupotomy intervention, standardized manual joint mobilization was performed under continuous axial traction. The clinician maintained a firm grasp on the distal humerus while executing controlled passive shoulder flexion, abduction, and extension to the patient’s maximum tolerated range. Audible crepitus, indicative of adhesion release, was observed during multi-directional stretching. Each position was held for 10 seconds with 3 repetition cycles to optimize tissue remodeling. Immediate posttreatment assessment demonstrated clinically significant improvements: passive range of motion increased to 150° abduction (representing a 115° gain from baseline), 130° forward flexion, and 30° extension, accompanied by reduction in VAS pain scores to 5 points (from preoperative 7 points), with these marked improvements documented in Figure 2.

Immediate post-procedural care included application of sterile gauze for hemostasis followed by adhesive bandage wound coverage, accompanied by 20-minute cryotherapy to the treated shoulder. Patients were instructed to avoid heavy lifting and excessive movement of the affected limb for 48 hours post-intervention. A structured rehabilitation protocol was implemented, consisting of: wall-climbing exercises (10 minutes/session, twice daily), resistance training (10 repetitions/set, 3 sets daily), and Codman pendulum exercises (5 minutes/session, thrice daily). Supplementary thermotherapy (40°C, 15 minutes/session) was prescribed to enhance tissue perfusion and promote healing.

The 6-week follow-up results showed that the active abduction of the left shoulder recovered to 160°, forward flexion to 150°, the VAS score was 2 points, and the difference in deltoid circumference was reduced to 0.5 cm. No recurrence was observed during the 6-month follow-up, and the patient’s activity of daily living scale (ADL) increased from 45 points before surgery to 85 points.

## 3. Discussion

The pathological hallmark of adhesive capsulitis lies in the fibrotic transformation of the glenohumeral joint capsule and biomechanical derangement of the periarticular kinetic chain. Contemporary medical studies^[8]^ have established that chronic nonbacterial inflammation triggers synovial hyperplasia and aberrant collagen deposition, leading to joint volume reduction, coracohumeral ligament contracture, and rotator interval adhesions – collectively forming a tripartite mechanical dysfunction involving the “joint capsule-tendon-bursa” complex. The compromised roll-glide mechanism of the glenohumeral joint and disrupted scapulothoracic rhythm further perpetuate the vicious cycle of pain-immobilization-atrophy. Current clinical management primarily relies on oral NSAIDs, intra-articular injections, and physical therapy, which provide transient anti-inflammatory effects but fail to address deep-seated fibrotic adhesions. While arthroscopic capsular release can restore joint mobility, it carries risks of postoperative capsular laxity and heterotopic ossification. Consequently, developing a therapeutic strategy that combines minimally invasive intervention with biomechanical reconstruction has emerged as a critical imperative for improving long-term outcomes in periarthritis management.

Adhesive capsulitis, from the perspective of traditional Chinese medicine’s meridian tendon theory, represents a concurrent pathological state of “tendon nodulation” and “articular misalignment.” In this case, occupational strain induced qi stagnation in the 3 yang meridians of the hand, leading to blood stasis accumulation at key acupoints including Jianzhen (SI9) and Naoshu (SI10), clinically manifesting as “meridian obstruction by blood stasis with tendon contracture and bone stiffness.” Acupotomy therapy exerts dual therapeutic effects through “qi regulation via needle stimulation” and “adhesion release via blade dissection,” achieving precise release of pathological structures including the thickened glenohumeral joint capsule, coracohumeral ligament, and periarticular contractile muscles. This directly interrupts pathological tension chains, consistent with the therapeutic principle articulated in *HUANG DI NEI JING*: “resolve accumulations and relax hypertonicity.”The therapeutic mechanisms can be systematically categorized into 3 aspects: anatomical Decompression: Targeted sectioning of fibrotic capsular regions and contracted ligaments expands glenohumeral joint space. Biomechanical rebalancing: simultaneous release of anterior tension muscles (pectoralis major and coracobrachialis) and posterior constrictors (latissimus dorsi and teres major) restores normal scapulohumeral rhythm (evidenced by 20°–25° improvement in dynamic scapular kinematics). Neurohumoral Modulation: Mechanical stimulation from acupotomy triggers local release of endogenous analgesics while enhancing microcirculation, thereby facilitating clearance of inflammatory metabolites.

The therapeutic efficacy of this case substantiates the scientific rationale of the multi-target release strategy. The immediate postoperative range of motion improvement resulted from mechanical adhesion release in the joint capsule, while the 6-week follow-up demonstrated restored rotator cuff functionality, closely associated with acupotomy-induced rehabilitation of the supraspinatus and mid-deltoid kinetic chains. Furthermore, postoperative wall-climbing exercises and resistance training reinforced scapular stabilizers (serratus anterior and lower trapezius), thereby consolidating biomechanical homeostasis. These outcomes collectively exemplify the integrated treatment philosophy of “structural intervention-functional rehabilitation,” where targeted anatomical release synergizes with progressive functional restoration to achieve sustained clinical improvement.

While demonstrating therapeutic efficacy, acupotomy treatment necessitates strict adherence to several key protocols: the procedure must rigorously follow the “neurovascular avoidance” principle, including limiting insertion depth to ≤3.0 cm at the coracoid process to prevent brachial plexus injury and maintaining ≤2.5 cm depth at the suprascapular nerve projection zone (supraspinatus insertion site); for cases with significant capsular calcification, adjunctive extracorporeal shockwave therapy is recommended to enhance release efficacy; postoperative rehabilitation protocols require further standardization, particularly in developing individualized training intensity and duration based on 3-dimensional shoulder motion analysis. Future research should employ dynamic ultrasonography to assess immediate post-acupotomy changes in capsular extensibility and utilize surface electromyography to quantitatively evaluate improvements in rotator cuff muscle coordination, thereby establishing predictive therapeutic models.

In summary, acupotomy therapy provides a structured therapeutic strategy – characterized by “minimally invasive adhesion release, dynamic stabilization, and functional rehabilitation” – for adhesive capsulitis through anatomically precise decompression and comprehensive biomechanical regulation. Its mechanism of action synergistically integrates traditional Chinese meridian tendon theory’s dynamic conceptualization of “tendon nodules” with contemporary biomechanical research on scapulohumeral chain coordination, demonstrating the unique advantages of integrative medicine in treating musculoskeletal disorders. Further advancements in multimodal image-guided target localization and the establishment of evidence-based indication grading systems will promote standardized application of this technique in shoulder pathology management.

## Acknowledgments

We thank all participants for their contributions to this study.

## Author contributions

**Conceptualization:** Zehao Hu, Qiuxuan Huang, Zhanxin Li.

**Data curation:** Zehao Hu, Qiuxuan Huang.

**Formal analysis:** Zehao Hu, Zhanxin Li.

**Investigation:** Qiuxuan Huang, Zhanxin Li.

**Methodology:** Zehao Hu, Qiuxuan Huang.

**Software:** Zehao Hu, Zhanxin Li.

**Supervision:** Zehao Hu, Zhanxin Li.

**Writing – original draft:** Zehao Hu, Qiuxuan Huang, Zhanxin Li.

**Writing – review & editing:** Zehao Hu, Qiuxuan Huang, Zhanxin Li.
